# Hooligans

**Published:** 1900-12-22

**Authors:** Honnor Morten


### Editor's Letter-Box.

[Our Correspondents are reminded that prolixity is a great bar to publication, and that brevity of style and conciseness of statement greatly facilitate early insertion.]

### HOOLIGANS.

Miss Honnor Morten writes :—If you spent a remarkable and interesting hour at St. Martin's Town Hall in hearing how to cure Hooligans by forgiveness, I have spent a most enjoyable ten minutes in reading your fresh and entertaining report of the proceedings. But, alas! my relations are not yet Socialists, and still believe there is something sacred about money, so I must ask you to correct the assertion that we used to steal my mother's money. It was cutlets and cheesecakes we used to raid—hiding on the stairs till the butler put them down in the hall, and then making a rush for them before the cook could whisk them off to the larder. I still fail to see much difference in the conduct of the boy who hides round a street corner till the old apple-woman is looking the other way and then snatches one of her apples. But the result is generally very different. Why?

